# Using GAM functions and Markov-Switching models in an evaluation framework to assess countries’ performance in controlling the COVID-19 pandemic

**DOI:** 10.1186/s12889-021-11891-6

**Published:** 2021-11-27

**Authors:** Abdinardo M. B. de Oliveira, Jane M. Binner, Anandadeep Mandal, Logan Kelly, Gabriel J. Power

**Affiliations:** 1 Department of Business, Federal University of Technology of Paraná, Paraná, Brazil; 2 Birmingham Business School, University of Birmingham, Birmingham, UK; 3 Department of Economics, University of Wisconsin, River Falls, USA; 4 Department of Finance, Insurance and Real Estate, Laval University, Laval, Canada

**Keywords:** COVID-19, Statistical models, Epidemiological monitoring

## Abstract

**Background:**

The COVID-19 pandemic has initiated several initiatives to better understand its behavior, and some projects are monitoring its evolution across countries, which naturally leads to comparisons made by those using the data. However, most “at a glance” comparisons may be misleading because the curve that should explain the evolution of COVID-19 is different across countries, as a result of the underlying geopolitical or socio-economic characteristics. Therefore, this paper contributes to the scientific endeavour by creating a new evaluation framework to help stakeholders adequately monitor and assess the evolution of COVID-19 in countries, considering the occurrence of spikes, "secondary waves" and structural breaks in the time series.

**Methods:**

Generalized Additive Models were used to model cumulative and daily curves for confirmed cases and deaths. The Root Relative Squared Error and the Percentage Deviance Explained measured how well the models fit the data. A local min-max function was used to identify all local maxima in the fitted values. The pure Markov-Switching and the family of Markov-Switching GARCH models were used to identify structural breaks in the COVID-19 time series. Finally, a quadrants system to identify countries that are more/less efficient in the short/long term in controlling the spread of the virus and the number of deaths was developed. Such methods were applied in the time series of 189 countries, collected from the Centre for Systems Science and Engineering at Johns Hopkins University.

**Results:**

Our methodology proves more effective in explaining the evolution of COVID-19 than growth functions worldwide, in addition to standardizing the entire estimation process in a single type of function. Besides, it highlights several inflection points and regime-switching moments, as a consequence of people’s diminished commitment to fighting the pandemic. Although Europe is the most developed continent in the world, it is home to most countries with an upward trend and considered inefficient, for confirmed cases and deaths.

**Conclusions:**

The new outcomes presented in this research will allow key stakeholders to check whether or not public policies and interventions in the fight against COVID-19 are having an effect, easily identifying examples of best practices and promote such policies more widely around the world.

**Supplementary Information:**

The online version contains supplementary material available at (10.1186/s12889-021-11891-6).

## Background

Pandemics and major epidemic outbreaks are not unlikely events, contrary to what common sense may imply. They are real threats. History tells us the effects on mankind of the Black Death in the 14th century and the Spanish flu in 1918. Over the past three decades, the number of reported outbreaks of highly pathogenic or highly transmissible infectious diseases has increased enormously [1, 2].

The number of deaths directly attributable to these outbreaks is not always large. However, a pandemic might have a catastrophic impact if it is not taken seriously, due to the non-linearity of its transmission in a world that is highly interconnected through long-range transportation [3, 4]. This is an ideal setting for the widespread transmission of COVID-19. As of September 15, 2021, more than 226 million people worldwide have been infected, and more than 4.1 million people have died since the first case was detected in December 2019 in China, according to data gathered by the Centre for Systems Science and Engineering [CSSE] at Johns Hopkins University [5, 6].

Several initiatives are conducting careful research worldwide to better understand the behavior of COVID-19, such as those modelling the reproductive ratio [7, 8], the mortality rate [9, 10], the influence of climatic variables [11, 12], and the short-run impact on the global economy [13, 14], whilst adopting the precautionary principle of averting the risk of ruin [4, 15].

In parallel, some projects are being undertaken to monitor the evolution of COVID-19 across countries [5, 16, 17], which naturally leads to comparisons made by those who use the data. Clearly, these “at a glance” comparisons may be misleading because the curve that should explain the evolution of COVID-19 is different across countries as a result of the underlying geopolitical or socio-economic characteristics.

To deal with this situation, [18] proposed a framework to monitor and evaluate the performance of public policies in confronting COVID-19 that are more/less efficient in the short/long term, employing a set of non-linear growth functions (exponential, logistic, Gompertz, Weibull, Richards) through a quadrants system. These functions are not highly accurate in the long run owing to spikes and “second waves” now evident in the COVID-19 data worldwide [19]. They predict only one inflection point, i.e., the global maximum and do not detect local maximum or minimum over time. The exception is exponential function, which goes to infinity and which may be anticipating a regime change in the COVID-19 series, and growth functions find it difficult to model such behavior.

This paper contributes to the scientific endeavor by creating a new evaluation framework to help stakeholders (policymakers, public sector health workers, resilience managers, and the general public) to:

1. Adequately monitor the evolution of reported confirmed cases and deaths in countries, considering the occurrence of spikes and “secondary waves”;
2. Identify structural breaks in the confirmed cases and deaths curves;
3. Assess the performance of their actions in the face of the spread of COVID-19.

We incorporate new evidence that pandemic fatigue is taking hold. This decrease in commitment to fighting the pandemic alters the behavior of the forecast errors present in the COVID-19 curves, causing a structural break in the variance of the residuals, or forecast errors. We are therefore able to use Markov-Switching models on the residuals of our forecasts to identify regime-switching in the COVID-19 time series. Our new methodology proves more effective in explaining the evolution of COVID-19 than growth functions worldwide, including highlighting several inflection points and regime-switching moments. Moreover, results from this research can be used by managers, for example, to provide an econometric justification for the prioritizing of vaccination programmes in the health care sector.

## Methods

### The generalized additive models

Generalized Additive Models (GAM) are generally regarded as a particular case of generalized linear models [20, 21]. These models use linear predictors which are themselves sums of smooth functions, e.g., polynomial, bin, running mean, among others, of predictor variables, where their basic building blocks are splines used to model relationships. This is the main difference from linear models: the latter use predictors directly in the model multiplied by a scalar [22]. The linear model is a special and limiting case of a GAM [23].

The GAM framework allows for the dependence of the response on the predictor variables to be specified flexibly. The model is, therefore, specified in terms of smooth, or basis, functions. However, to obtain this convenience and flexibility, it is necessary to:

- Determine an appropriate representation for the smooth functions;
- Choose how smooth they should be.

A GAM, in its simplest form, can be represented as the Eq. 1, where *y*_*t*_ is a response variable, *x*_*t*_ is a predictor variable, *b*_*j*_(*x*) is a basis function as described above, *β*_*j*_ are the unknown coefficients, *k* is the basis dimension, which controls the degree of the model smoothness, the number of knots in a basis function, and it is part of model specification, and *ε*_*t*_ is a zero-mean, i.i.d. random variable.

1 $$ y_{t} = \sum_{j=1}^{k}b_{j}(x)\beta_{j} + \epsilon_{t}   $$

Thus, Eqs. 2 and (3) show the GAM functions for confirmed cases and deaths from COVID-19, respectively, with $\zeta _{t} \thicksim N(0,\sigma ^{2})$ random variables.

2 $$\begin{array}{@{}rcl@{}} {confirmed}_{t} &=& \sum_{j=1}^{100}b_{j}({day}_{t})\beta_{j} + \zeta_{t} \end{array} $$

3 $$\begin{array}{@{}rcl@{}} {deaths}_{t} &=& \sum_{j=1}^{100}b_{j}({day}_{t})\beta_{j} + \zeta_{t} \end{array} $$

These equations do not directly calculate the occurrence of the inflection points, however: these are the moments when the growth rate is going to decrease. This is common when using growth functions [24] to investigate outbreaks and epidemics [2, 18, 25]. To counter this limitation, Eqs. 4 and (5) show the GAM functions for daily cases and deaths from COVID-19.

4 $$\begin{array}{@{}rcl@{}} {dailycases}_{t} &=& \sum_{j=1}^{50}b_{j}({day}_{t})\beta_{j} + \varsigma_{t} \end{array} $$

5 $$\begin{array}{@{}rcl@{}} {dailydeaths}_{t} &=& \sum_{j=1}^{50}b_{j}({day}_{t})\beta_{j} + \varsigma_{t} \end{array} $$

Using time series of daily or deaths cases, unlike cumulative series, it is possible to estimate their smoothed curves. These curves approximate their growth rate functions, which allow us to identify their inflection points. In this case, we set *k* = 50 to increase the degree of smoothness, using $\varsigma _{t} \thicksim Pois(\lambda)$ i.i.d. random variables. Recall: the lower the *k* values, the smoother the fitted curve is, which helps to find the most relevant inflection points, the local maxima. Otherwise, the fitted curve would be very wiggly, making all the points local minima and maxima.

The coefficients of Eqs. (1–5) are estimated by restricted maximum likelihood (REML) as a bias-reducing alternative to maximum likelihood (ML), given that the latter tends to underestimate the variance components. Moreover, compared with the generalized cross-validation (GCV) estimator, REML tends to be more resistant to occasional severe over-fitting. Its optimum tends to be more pronounced relative to sampling variability, and it has less tendency to develop phantom minima when there is no real signal in the data, with an *O*(*n*^−4/5^) computational cost [21, 26].

To measure how well the models fit the data, the Root Relative Squared Error (RRSE) was used for Eqs. (2–3), while the percentage deviance explained was used for Eqs. (4–5). The percentage deviance explained, which is a generalization of *R*^2^, is based on the sum of squares of the deviance residuals, as the fitted model deviance, divided by the sum of squares of the deviance residuals when the covariate effects are set to zero, as the null deviance [21]. The higher the values, the better.

The local.min.max function from the spatialEco R package [27] was used to identify all local maxima or peaks in the fitted values, also known as inflection points. This method is simpler to explain and more straightforward than simulating multivariate normal random deviates, as proposed by [28].

### The Markov-Switching structural break functions

When using Eqs. (2–3), we assumed that the residuals follow a normal distribution, with a constant variance: $\zeta _{t} \thicksim N(0,\sigma ^{2})$.

It appears, however, that the occurrence of a new more contagious wave of COVID-19 is spreading faster than the first outbreak in spring 2020 according to top scientists [19, 29]. Member States across the WHO European Region are reporting emerging pandemic fatigue in their populations. Pandemic fatigue is an expected and natural reaction to the prolonged nature of this crisis and the associated inconvenience and hardship. It poses a serious threat to efforts to control the spread of the virus, see [30] for a Policy framework for reinvigorating the public to prevent the pandemic.

In the same vein,[19] provides evidence that people relaxed their commitment to non-pharmacological measures to combat COVID-19: mask-wearing, hand-washing, and social distancing after the first wave of contagion. Furthermore, [31] showed that social distancing can result in an estimated 65% reduction in new COVID-19 cases, while [32], using the situation in Manitoba, Canada as an example, verified that relaxing social distancing to levels of contact that are 50% of what they were before COVID-19 may result in over 35% of the population infected at the same time. Both studies corroborate our contention, reinforced by [30], that pandemic fatigue is taking hold.

This decrease in commitment to fighting the pandemic alters the behavior of the forecast errors present in the COVID-19 curves, causing a structural break in the variance of the residuals, or forecast errors. We are particularly looking for this phenomenon in the residuals because the lower the RRSE, the greater the possibility of the residuals of the series being stationary, thus allowing, for instance, the use of the Markov-Switching (MS) models.

The main features of the MS models are:

1. The regime that occurs at time t is determined by an unobservable random process, $S^{i}_{t}$;
2. Each regime is assumed to be a first-order Markov process, that is, the current regime only depends on the previous one [33].

The Lamoureux and Lastrapes test [34] was used to verify the occurrence of structural breaks in the variance of the residuals in equations (2-3). The test consisted of estimating the *α* and *β* coefficients of a GARCH model (1,1) applied to standardized residuals. If the sum of these coefficients is very close to 1, the existence of structural breaks in the residuals is confirmed, which implies the existence of at least two regimes. This finding suggests that a change in people’s behavior might be contributing to a greater increase in infected people, as pointed out by [19].

The next step was to select the best approach to represent the structural breaks in the residuals of Eqs. (2–3). For simplicity of modeling these approaches, we assume the existence of two regimes: low (high) variance, which represents a stronger (weaker) commitment to non-pharmacological measures to combat COVID-19. Our first approach is to consider a pure Markov-Switching (MSwM) model of variance, as proposed by [35]. Here the persistence in the variance (previous values keep affecting posterior values) occurs due to the regime-switching of the variance process. This model can be described using the following set of equations and definitions:

6 $$ \begin{aligned} \zeta_{t} \thicksim N(0,\sigma^{2}) \end{aligned}  $$

7 $$ \begin{aligned} {}\sigma^{2}_{t} = \sigma^{2}_{1}S_{1t} + \sigma^{2}_{2}S_{2t} \end{aligned}  $$

8 $$ \begin{aligned} {}\sigma^{2}_{1} < \sigma^{2}_{2} \end{aligned}  $$

9 $$ \begin{aligned} {} S_{kt} = 1, if S_{t} = k; otherwise S_{kt} = 0, k = 1,2 \end{aligned}  $$

10 $$ \begin{aligned} {}p(S_{t} = 1 | S_{t-1} = 1) = p_{11};p(S_{t} = 2 | S_{t-1} = 1) = 1 - p_{11} \end{aligned}  $$

11 $$ \begin{aligned} {}p(S_{t} = 2 | S_{t-1} = 2) = p_{22};p(S_{t} = 1 | S_{t-1} = 2) = 1 - p_{22} \end{aligned}  $$

12 $$ {}\begin{aligned} L(\epsilon,\theta) = \sum_{t=1}^{T}\sum_{i=1}^{2} \frac{p_{ii}}{\sqrt{2\pi\sigma^{2}_{i}}}\exp\left\{\frac{-\left(\epsilon_{t} - \mu_{i} \right)^{2}}{2\sigma^{2}_{i}} \right\}  \end{aligned}  $$

where the vector of parameters $\theta \equiv \left \{\mu _{1},\mu _{2},\sigma ^{2}_{1}, \sigma ^{2}_{2}, p_{11},p_{22} \right \}$ can be estimated by a log-likelihood function in Eq. 12 using numerical methods [36]. With *p*_11_ and *p*_22_, it is possible to construct the transition matrix, essential to calculate the 1-step ahead regime. We used the MSwM R package [37] to estimate the vector *θ*. When using this approach, it is important to highlight that we assume that the variance remains constant within each regime.

The second approach considers the family of Markov-Switching GARCH models [38], where the variance can be time-varying in each regime (*k*=1,2). This means that the persistence in the variance occurs both for the shocks and the regime-switching in the parameters of the variance process.

We used the set of equations and conditional distributions (with zero mean and unit variance) available from the MSGARCH R package, as shown in Tables 1 and 2, respectively [45]. Thus, it is possible to estimate up to 30 types of models.

13 $$ L\left(\psi,I_{t}\right) = \prod_{t=1}^{T}f(\epsilon_{t}|\psi,I_{t-1})   $$

**Table 1:** MSGARCH models

Label	Equation	Author
“sARCH”	$\sigma ^{2}_{k,t} = \omega _{k} + \alpha _{k}\epsilon ^{2}_{t-1}$	Engle [39]
“sGARCH”	$\sigma ^{2}_{k,t} = \omega _{k} + \alpha _{k}\epsilon ^{2}_{t-1} + \beta _{k}\sigma ^{2}_{k,t-1}$	Bollerslev [40]
“eGARCH”	$\ln \left (\sigma ^{2}_{k,t}\right) = \omega _{k} + \alpha _{1,k}\left (\left |\eta _{k,t-1}\right | - E\left [\left |\eta _{k,t-1}\right | \right ]\right) + $	Nelson [41]
	$+ \alpha _{2,k}\eta _{k,t-1}+\beta _{k}\ln \left (\sigma ^{2}_{k,t-1}\right)$	
“gjrGARCH”	$\sigma ^{2}_{k,t} = \omega _{k} + \left (\alpha _{1,k}+\alpha _{2,k}I \left \{\epsilon _{t-1}<0\right \} \right)\epsilon ^{2}_{t-1} + $	GJR [42]
	$+\beta _{k}\sigma ^{2}_{k,t-1}$	
“tGARCH”	$\sigma _{k,t} = \omega _{k} + \left (\alpha _{1,k}I \left \{\epsilon _{t-1}\geqq 0\right \} - \alpha _{1,k}I \left \{\epsilon _{t-1}\geqq 0\right \}\right) \epsilon _{t-1}+$	Zakoian [43]
	+*β*_*k*_*σ*_*k*,*t*−1_

**Table 2:** MSGARCH conditional distributions

Distribution	Equation	Label
Normal	$fN(\eta) = \frac {1}{\sqrt {2\pi }}\exp \left (-\frac {\eta ^{2}}{2}\right)$	“norm”
Student-t	$fS(\eta ;\nu) = \frac {\Gamma \left (\frac {\eta + 1}{2} \right)}{\sqrt {\left (\nu -2\right)\pi }\Gamma \left (\frac {\nu }{2}\right)} \left (1 + \frac {\eta ^{2}}{\left (\nu -2\right)}\right)^{-\frac {\nu +1}{2}} $	“std”
GED	$f_{GED}(\eta ;\nu) \equiv \frac {\nu \exp \left (-\frac {\left |\eta /\lambda \right |^{\nu }}{2} \right)}{\lambda 2^{\left (1+1/\nu \right)}\Gamma (1/\nu)}, \lambda \equiv \left (\frac {\Gamma (1/\nu)}{4^{1/\nu }\Gamma (3/\nu)} \right)^{1/2} $	“ged”
Skewed Normal	See Trottier and Ardia [44], equation 1	“snorm”
Skewed Student-t	See Trottier and Ardia [44], equation 1	“sstd”
Skewed GED	See Trottier and Ardia [44], equation 1	“sged”

Let *ψ* be the vector of model parameters. The likelihood function is defined in Eq. 13, and the maximum likelihood estimator $\widehat {\psi }$ is obtained by maximizing the logarithm of (13), where *f*(*ε*_*t*_|*ψ*,*I*_*t*−1_) denotes the density of *ε*_*t*_ given past observations, *I*_*t*−1_ and model parameters *ψ*.

## Results

### Use of GAM functions to predict COVID-19 historical series

**Fig. 1:**
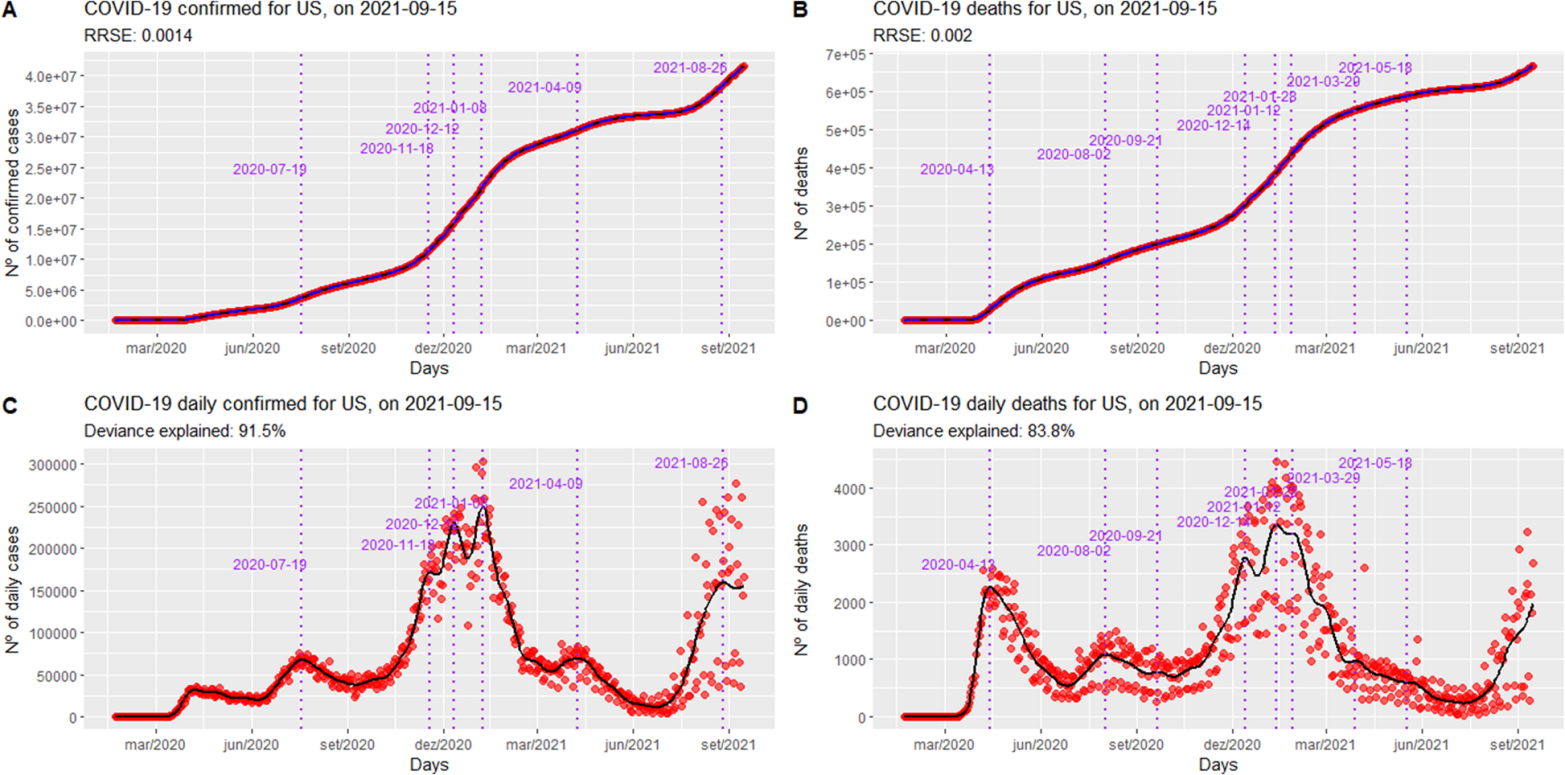
COVID-19 curves for the USA, as of September 15, 2021. Panel A shows cumulative confirmed cases; panel B shows cumulative deaths; panel C shows daily confirmed cases, and panel D shows daily deaths

Countries’ data were collected using *tidycovid19* R package [6]. The results for COVID-19 curves in the USA are shown in Fig. 1 as an example of the modeling proposed by Eqs. (2–5), respectively. The red points are the actual values, the black solid lines are the fitted values, the dashed blue lines are the 99% forecasting intervals, and the vertical dashed purple lines are the inflection points.

**Fig. 2:**
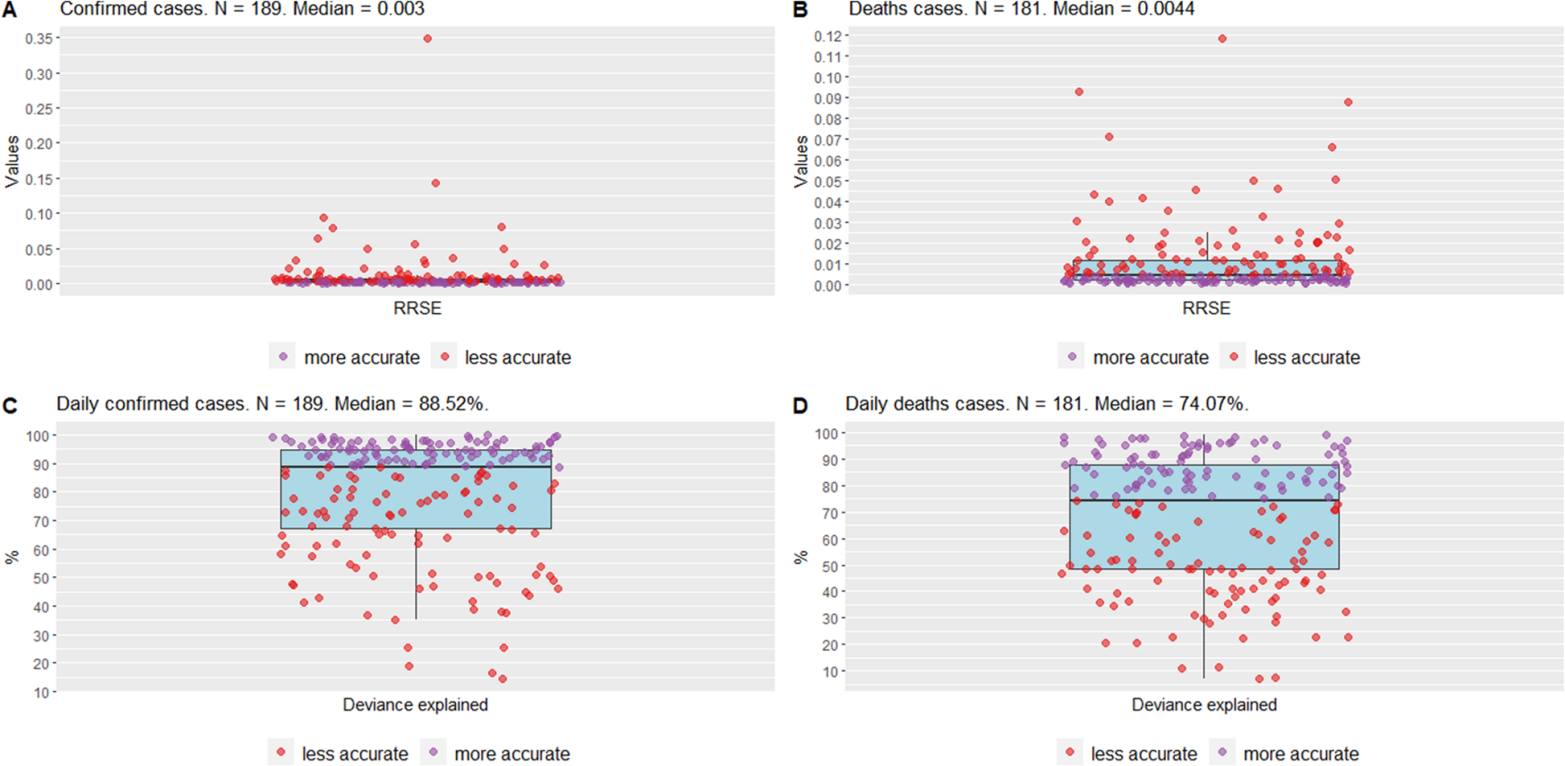
Boxplots for RRSE and Deviance explained worldwide, on September 15, 2021. Panel A shows cumulative cases; panel B shows cumulative deaths; panel C shows daily cases, and panel D shows daily deaths

Figure 2 shows the results of Eqs. (2–5) applied to several countries worldwide, represented by purple/red dots. The median of the RRSE shows that the models fit the data well, both for confirmed cases and deaths (A-B). Moreover, when countries are classified as “less accurate”, that is, the RRSE is above the median, the maximum RRSE is close to 0.15, except in one case, which is considered to be an outlier.

Similarly, the panels in Fig. 2 reporting results for deviance explained also suggest a good fit for Eqs. (4–5) to smooth the daily cases and death time series for the countries, given that their medians are above 74% (the higher, the better).

As a comparison, we considered several growth functions (exponential, logistic, Gompertz, Weibull, and Richards) [24] to fit the confirmed cases of the countries. Next, we computed their RRSE. To estimate the growth functions parameters, we based our estimation on the previous study by [18], choosing the function that best fits each country using RRSE.

**Fig. 3:**
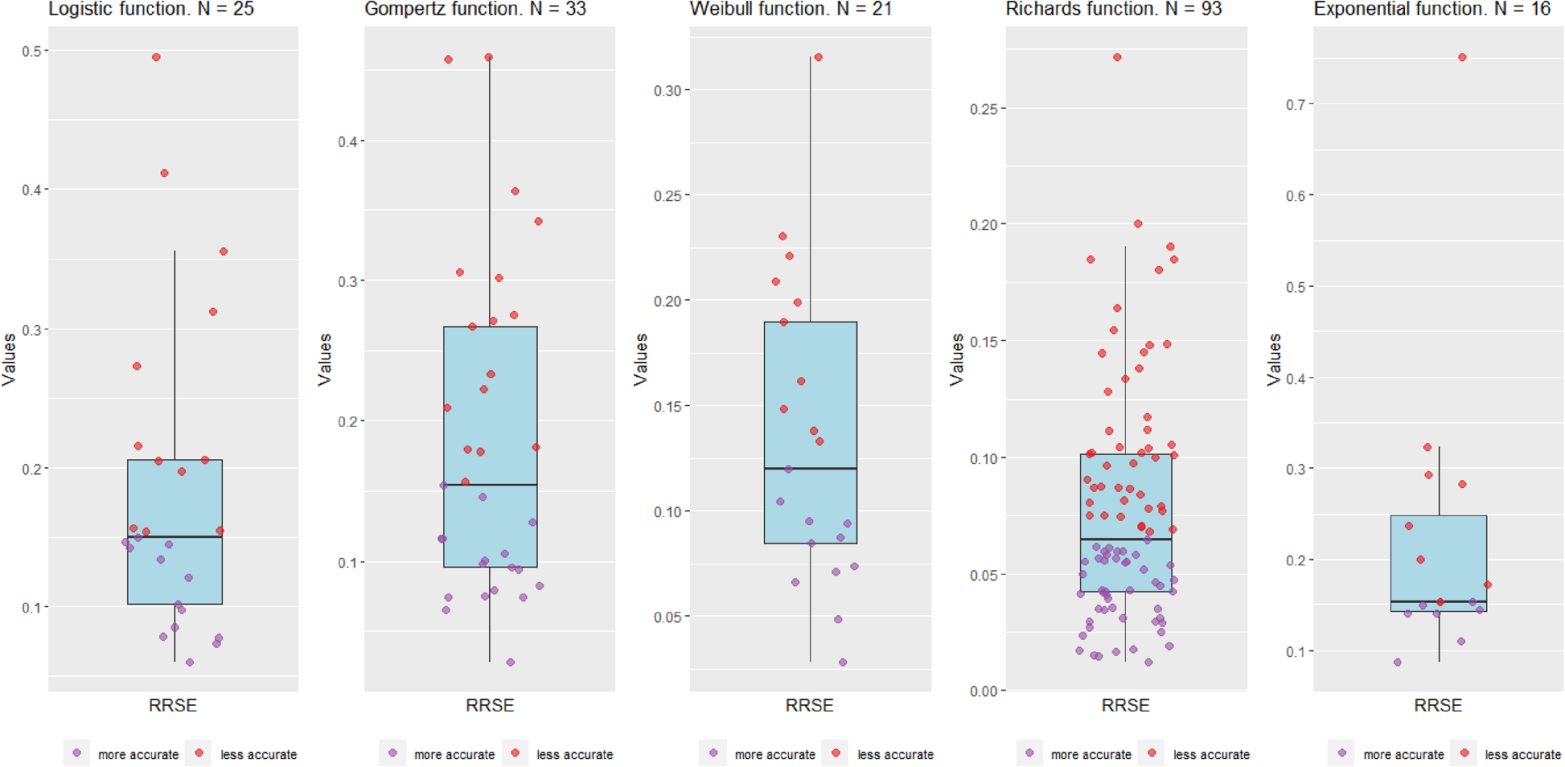
Boxplots for RRSE of growth functions worldwide on September 15, 2021

Figure 3 shows the RRSE for each of the five growth functions considered, for the confirmed cases, given that [18] analyzed only this time series. Our results clearly show that the GAM functions outperform those of [18] for the fitting of the COVID-19 time series, in addition to standardizing the entire estimation process in a single type of function.

### Identifying structural breaks in the COVID-19 curves

**Fig. 4:**
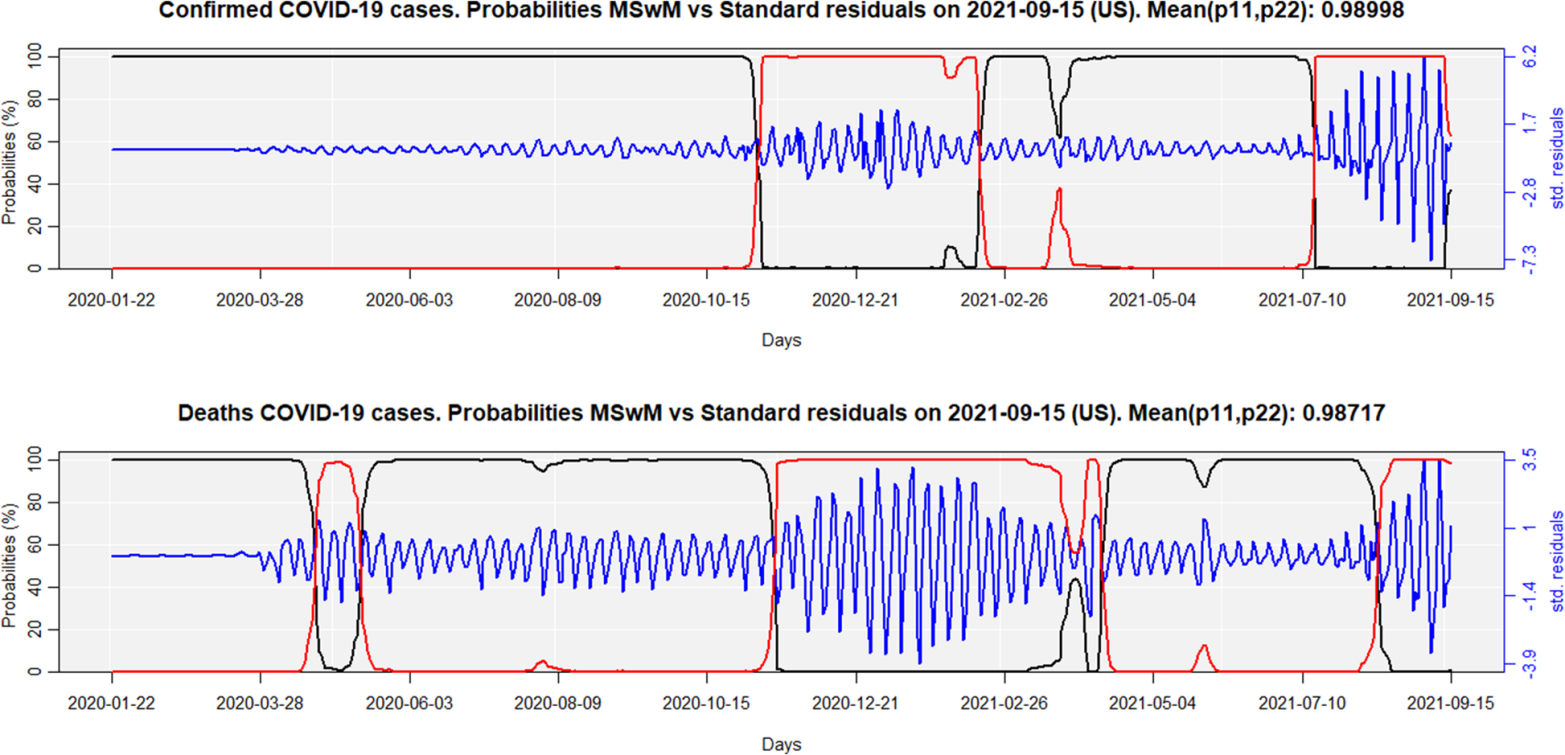
MSwM probabilities for confirmed cases and deaths, linked to COVID-19 for the USA

Continuing with the COVID-19 for the USA series as an example, the GARCH (1,1) [$\sigma ^{2}_{t} = \omega + \alpha \epsilon ^{2}_{t-1} + \beta \sigma ^{2}_{t-1}$] coefficients were estimated for the residuals of Eqs. (2–3) using the rugarch R package [46], assuming a Normal distribution, as illustrated in Table 3, with 1% of significance. In both models, the sum of the *α* and *β* coefficients is equal to 1, confirming the existence of a structural break in the residuals, as shown in Figure 4. In other words, the parameters of the variance’s residuals change over time.

**Table 3:** GARCH models result for residuals for the USA, on September 15, 2021

GARCH model (USA)	*ω*	*α*	*β*
Residuals from Eq. 2	0.00000	0.32943	0.67057
Residuals from Eq. 3	0.00005	0.29769	0.70231

Figure 4 shows the results for the pure Markov-Switching models (MSwM) for the confirmed cases and deaths from COVID-19 in the USA, on September 15, 2021. The black and red lines are the probabilities of being, respectively, in the regime of low (*σ*_1,*confirmed*_=0.32;*σ*_1,*deaths*_=0.48) and high (*σ*_2,*confirmed*_=1.85;*σ*_2,*deaths*_=1.59) variance/std. deviation, while the blue line is the standardized residuals.

Regarding confirmed cases, the MSwM models indicate that a regime-switching started on November 8th, 2020 (five days after the United States presidential election), from low variance to high variance. On February 16, 2021, the USA returned to the low variance regime since they administered the first vaccine on December 14, 2020 [47]. However, on July 17, 2021, they came again to the high variance regime, mainly due to the proliferation of the delta variant in the country, together with the decrease in the effectiveness of vaccines against this new variant of COVID-19 [48]. As for the number of deaths, the regime-switching from low variance to high variance, and vice-versa, follows a pattern similar to that observed for confirmed cases.

**Fig. 5:**
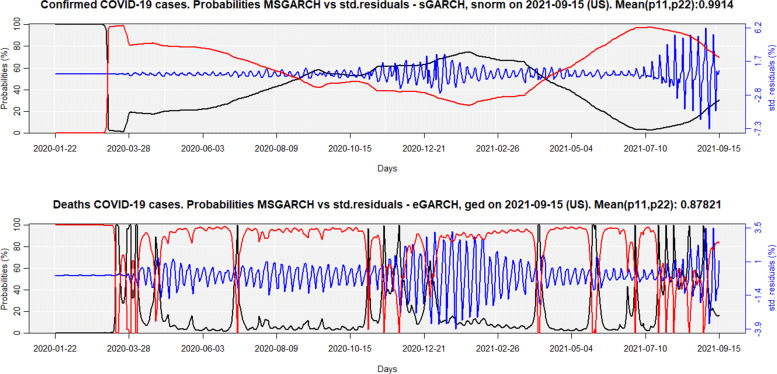
MSGARCH probabilities for confirmed cases and deaths, linked to COVID-19 for the USA

Figure 5 shows the results for the Markov-Switching GARCH models (MSGARCH) for the COVID-19 curves, for confirmed cases and deaths in the USA, as of September 15, 2021. As in Fig. 4, the black and red lines are the probabilities of being in the regime of low and high variance/std. deviation, while the blue line shows standardized residuals.

Given that 30 models were estimated for both curves, the choice of the best model was based on the following criteria: all model parameters must be significant at the 5% level, and the best model must have the lowest Bayesian Information Criteria [BIC]. The estimated coefficients for the best models are shown in Table 4, for confirmed cases and deaths.

**Table 4:** MSGARCH estimated parameters for the USA COVID-19 curves

sGARCH-snorm	Confirmed cases		eGARCH-ged	Deaths	
	Low var	High var		Low var	High var
*ω*_1_	0.0000	0.0001	*ω*_1_	0.1019	-0.0290
*α*_1_	0.2502	0.1431	*α*_1_	0.3636	0.3244
*β*_1_	0.7492	0.8567	*α*_2_	-0.1452	0.1729
*ξ*_1_	1.3930	1.4308	*β*_1_	0.9621	0.9969
-	-	-	*ν*_1_	4.8038	5.9520

Both Fig. 4 and 5 show the mean of the probabilities *p*(*S*_*t*=1_|*S*_*t*−1_=1)=*p*_11_ and *p*(*S*_*t*_=2|*S*_*t*−1_=2)=*p*_22_ for confirmed cases and deaths, from their transition matrices. The higher these values, the more persistent is the regime. Otherwise, the regime-changing would be easier, thus making no sense for the use of Markov-Switching models, and a single regime GARCH model could better explain the low/high variability of the residuals. Therefore, the best model is that with the highest *mean*(*p*_11_,*p*_22_), suggesting that MSGARCH slightly better explains the regime-switching for confirmed cases, while the MSwM better explains deaths.

**Fig. 6:**
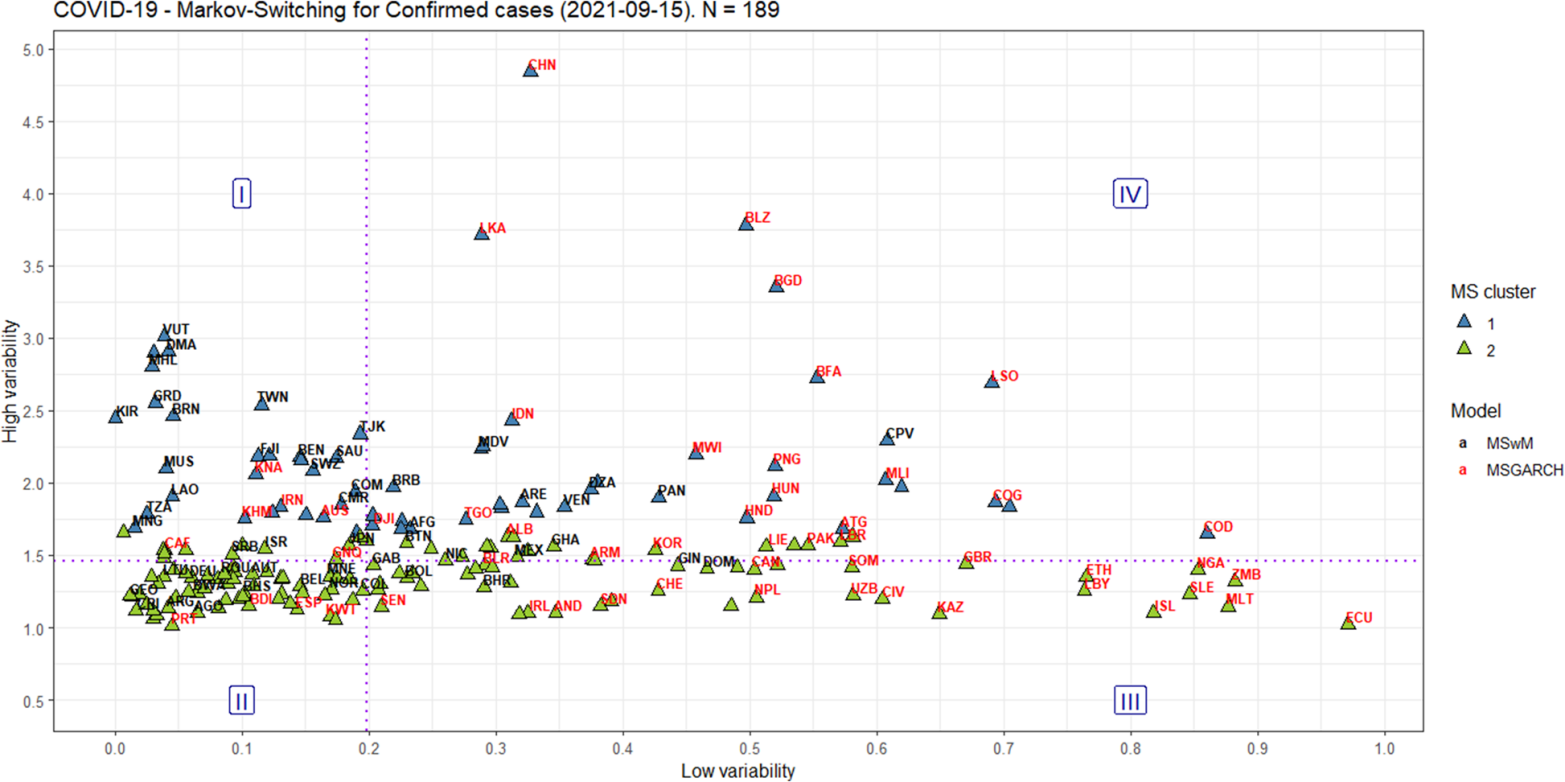
Markov-Switching classification for the confirmed cases worldwide

**Fig. 7:**
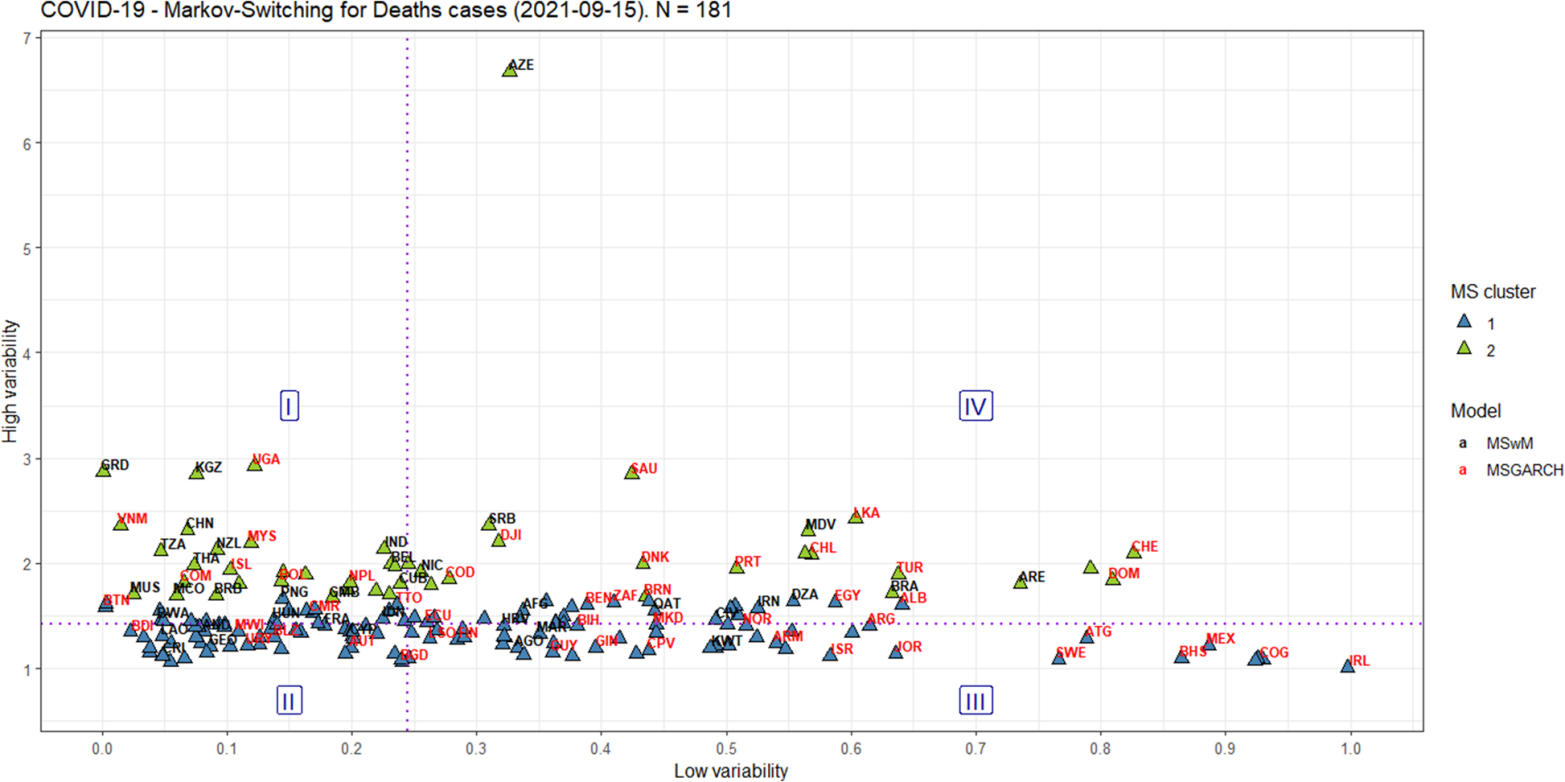
Markov-Switching classification for the deaths worldwide

Figures 6 and 7 display the classification of the Markov-Switching model that better explains the structural breaks amongst countries, on September 15, 2021, following the above-mentioned criteria of choice. For the confirmed cases and deaths, the MSwM accounts for 96 and 83 countries, respectively, while the MSGARCH accounts for 93 and 98 countries, in that order.

When the MSGARCH is chosen, the eGARCH-ged model is selected 12 out of 93 times for the confirmed cases, and the eGARCH-sged and eGARCH-snorm models are selected 14 out of 98 times for the deaths, being those models their modes, respectively. It is essential to highlight that the variance in the eGARCH model respond asymmetrically to rises and falls in COVID-19 numbers, determined by the *α*_2_ parameter. In the USA example, for deaths, the variance increases when the residual is positive (*α*_2_>0) for the high regime, while when the regime is low, the variance increases when the residual is negative (*α*_2_<0).

Besides, we can add two other possibilities of classification for the countries regarding the occurrence of structural breaks in the confirmed cases and deaths from COVID-19 curves. Again, we contend that pandemic fatigue causes a change in people’s behavior and contributes to a greater increase in infected people, as pointed out by [19].

The first possibility was using the Partitioning Around Medoids (PAM) [49], given that it uses the K-medoid algorithm, which is a robust alternative to the K-means algorithm because it is less sensitive to noise and outliers. Moreover, it employs the silhouette method to find the optimal k clusters over a range of possible values, because it measures the quality of clustering: the higher, the better, in a scale of Average Silhouette Width (ASW), ranging from zero to one. We define 01 to 10 as the range of possible values for k, and we found two clusters as the best number of clusters for the confirmed cases (ASW=0.48) and deaths (ASW=0.49).

The second possibility was to define quadrants from the medians of the axes, equally splitting the countries’ sample per axis, and dividing the Cartesian plane into four regions (I-IV), interpreted in a counter-clockwise direction. Region I is defined as having small “low-variability”/great “high-variability” [confirmed=41; deaths=46], region II as having small “low-variability”/small “high-variability” [confirmed=54; deaths=45], region III as having great “low-variability”/small “high-variability” [confirmed=41; deaths=46], and region IV as having great “low-variability”/great “high-variability” [confirmed=53; deaths=44].

Overall, on September 15, 2021, Figs. 6-7 show how heterogeneous the countries were concerning the commitment of their populations to non-pharmacological measures to combat COVID-19. Countries in Region II seem to have the best performance in this regard.

### A proposal for the COVID-19 evaluation framework

**Fig. 8:**
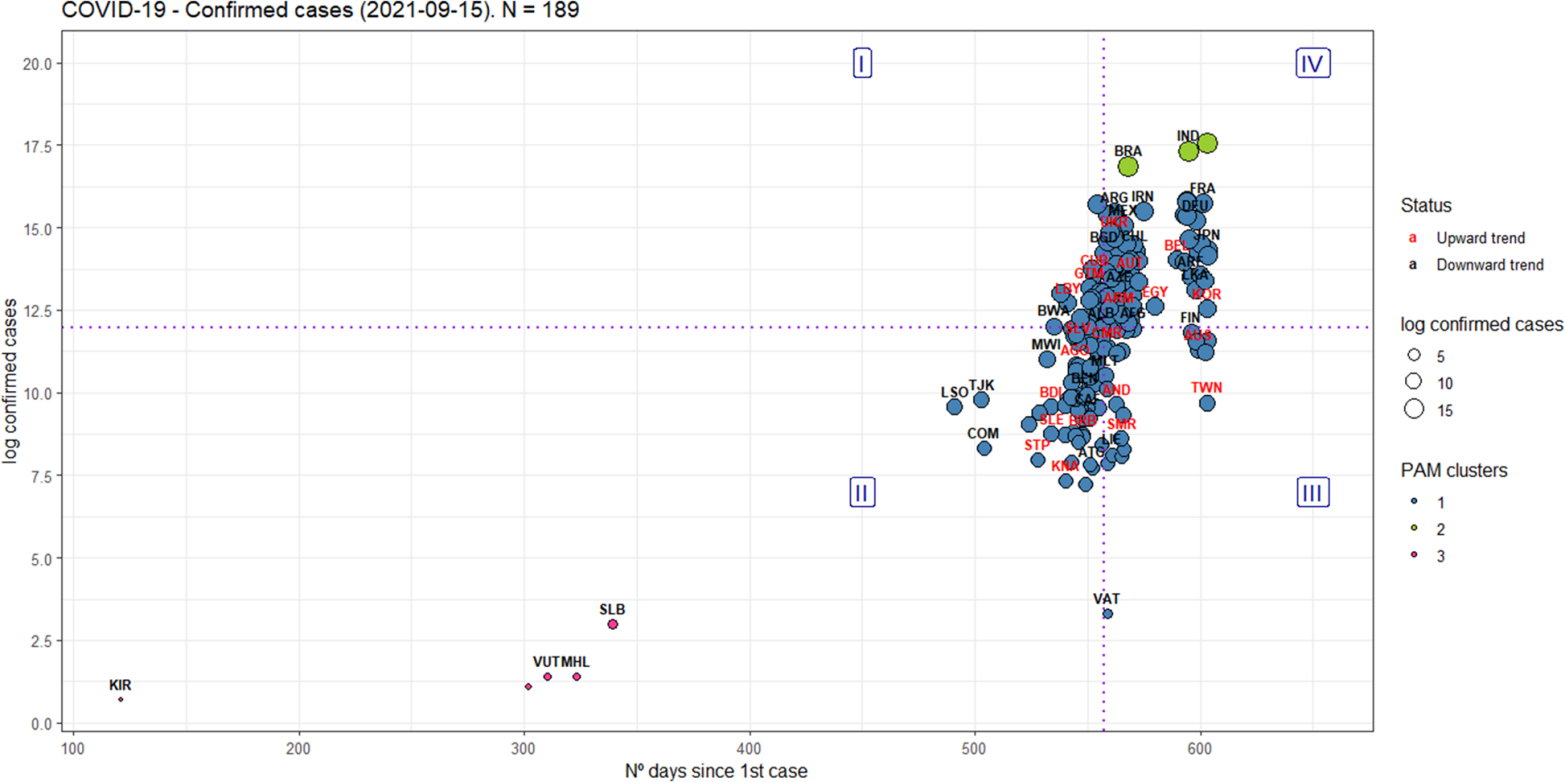
An evaluation framework for the confirmed cases worldwide

**Fig. 9:**
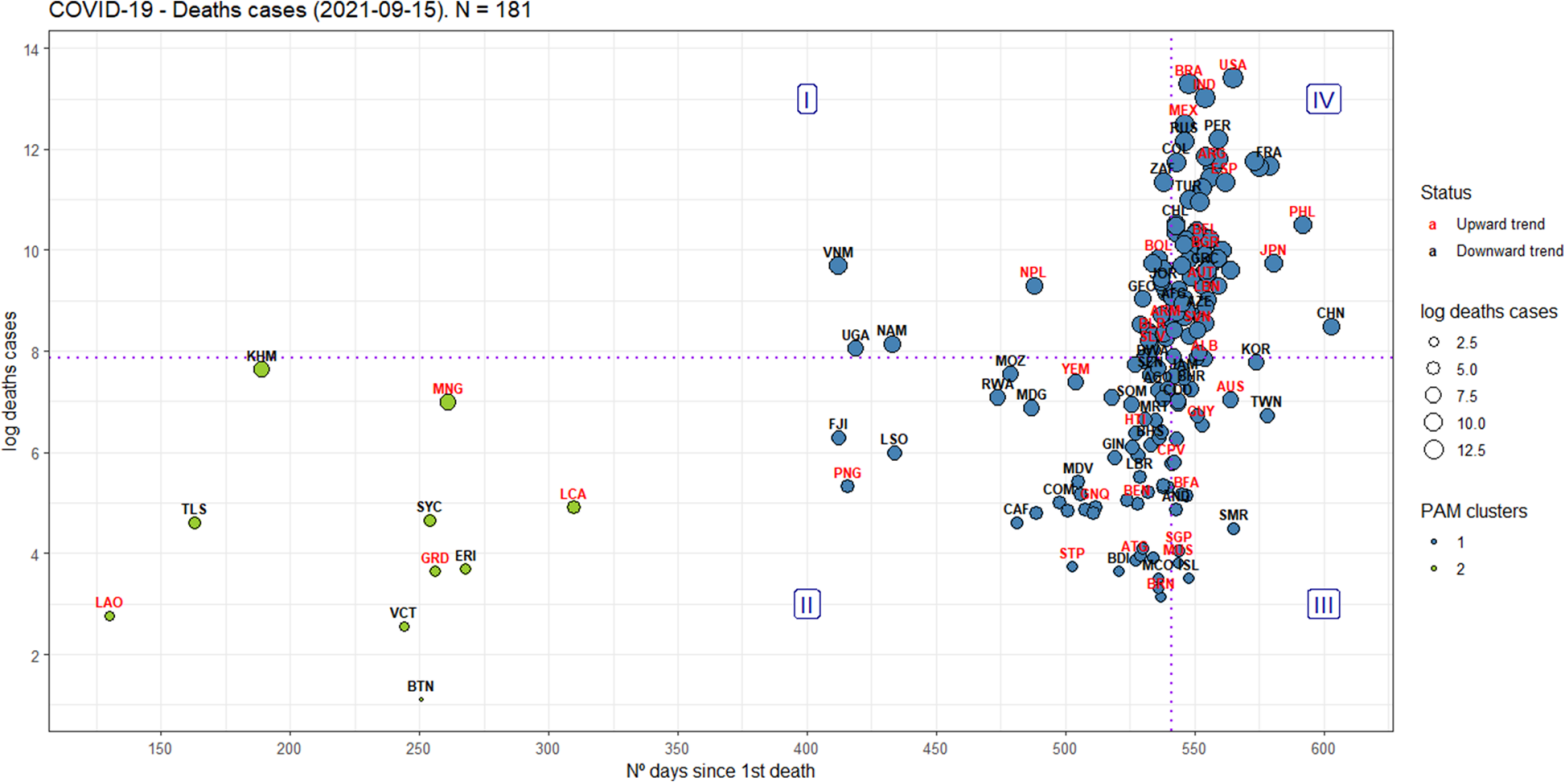
An evaluation framework for the deaths worldwide

In Figs. 8-9, the x-axis represents the cumulative number of days since the first COVID-19 case, and the y-axis is the natural logarithm of the current number of COVID-19 confirmed cases or deaths, as well as the size of the circles for each country, to facilitate a relative comparison between them: the bigger, the worst. To mediate the relationship between them, one more variable is considered: the inflection point.

Like Figs. 6, 7, 8 and 9 were divided into quadrants from the medians of the axes, equally splitting the countries’ sample per axis, and dividing the Cartesian plane into four regions (I-IV), also interpreted in a counter-clockwise direction.

Region I is defined as having “short-term inefficiency” [confirmed=22; deaths=26], region II as having “short-term efficiency” [confirmed=73; deaths=67], region III as having “long-term efficiency” [confirmed=22; deaths=24], and region IV as having “long-term inefficiency” [confirmed=72; deaths=64].

Therefore, the key concept of efficiency here is interpreted as preventing the number of confirmed cases and deaths from increasing over time, as well as to see, in the same chart, how the behavior of the growth rate.

Moreover, using the PAM clustering [49], clusters were also found in Figs. 8-9, thus showing heterogeneity in their performance, even in the same quadrant (ASW=0.89 for the confirmed cases, ASW=0.81 for the deaths).

## Discussion

In the Background section, we mentioned the role of geopolitical and socio-economic characteristics in explaining the evolution of COVID-19. However, it is also important to comment the role of psychological effects on people caused by the spread of COVID-19 around the world.

For example, several studies have assessed the fear of healthcare professionals or medical students of being infected with COVID-19, as well as how this fear affects their physical, mental, and emotional health [50–53]. Among the various results obtained using the Fear of COVID-19 Scale [54], women are more afraid than men of being infected by COVID-19, causing greater impacts on the quality of their physical, mental and emotional health. Perhaps, for these reasons, women, more than men, are also more likely to adopt non-pharmacological prevention measures.

Therefore, our evaluation framework also allows us to conjecture that countries classified as efficient, whether in the short or long term, also have the highest levels of the physical, mental, and emotional quality of their health professionals or medical students. The importance of this is that these professionals work on the front line in the fight against COVID-19.

**Fig. 10:**
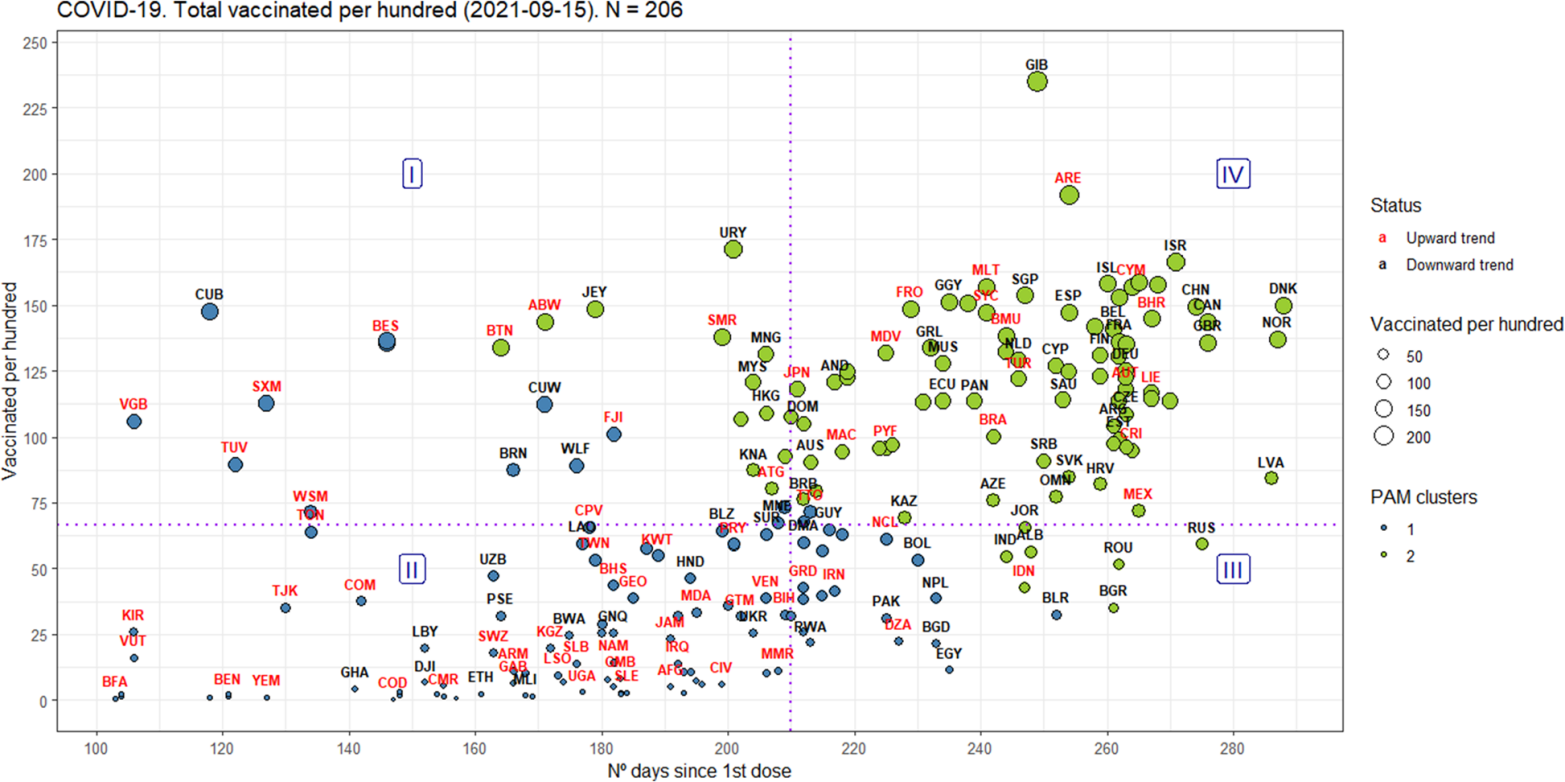
An evaluation framework for the total vaccinated per 100 worldwide

Although not the main focus of this study, our evaluation framework can also be applied to assess the vaccination deployment worldwide, in order to contribute to the perception of vaccine safety and increase willingness to receive it, as pointed out by [55]. Recently, [56] have made available a free-to-access global dataset that tracks the scale and rate of vaccine rollout. For instance, Fig. 10 shows, on September 15, 2021, the distribution of countries for the total vaccinated per hundred, since the 1st dose applied. In this case, countries in regions I and IV are classified as “short-term efficiency” and “long-term efficiency”, respectively.

Finally, on September 15, 2021, the results presented in Figs. 8–9 for the countries can be summarized as follows, considering the regions as defined in the World Bank’s Development Indicators:

1. For the confirmed cases, 105 out of the 189 countries showed a growth rate on a downward trend;
2. For the deaths, 99 out of the 181 countries showed a growth rate on a downward trend;
3. For confirmed cases and deaths, most of the countries on a downward trend are located in Sub-Saharan Africa, Europe & Central Asia. However, most of the countries on an upward trend are located in Europe & Central Asia, Sub-Saharan Africa, and Latin America & Caribbean, in this order;
4. For the confirmed cases, most of the countries considered efficient (quadrants II and III) are located in Sub-Saharan Africa (42), East Asia & Pacific (16), and Latin America & Caribbean (16). On the other hand, most of the countries considered inefficient (quadrants I and IV) are located in Europe & Central Asia (38), Latin America & Caribbean (17), and Middle East & North Africa (17);
5. For the deaths, most of the countries considered efficient (quadrants II and III) are located in Sub-Saharan Africa (39), Europe & Central Asia (17), and Latin America & Caribbean (13). On the other hand, most of the countries considered inefficient (quadrants I and IV) are located in Europe & Central Asia (34), Latin America & Caribbean (18), and Middle East & North Africa (13);
6. The United States, India, and Brazil have the highest confirmed cases among all countries, but only the USA has its growth rate on a upward trend;
7. Regarding the deaths, The United States, Brazil, India, Mexico and Peru have the highest number of victims among all countries, but Peru have its growth rate on an downward trend;

The figures mentioned above from our evaluation framework show that the most developed countries are not necessarily the most efficient in combating COVID-19. Europe is the most developed continent in the world and is home to 4 of the 7 members that constitute the G7, but most countries are trending upwards and considered inefficient, for confirmed cases and deaths.

Furthermore, even though 43.3% of the world’s population has received at least one dose of the COVID-19 vaccine [56], Figs. 8 and 9 illustrate that several countries are again facing waves of contagion, including those that were pioneers in vaccinating their populations, like Israel [57]. Another example comes from Figure 1: the USA is experiencing a new wave of infections and deaths similar to what they experienced in mid-December/2020 when they started their vaccination campaign against COVID-19.

## Conclusion

The purpose of this paper is to propose a new framework to monitor and assess, daily, the performance of countries in the fight against COVID-19. Our process will provide a greater understanding by stakeholders (policy-makers, public health workers, and the general public) of the evolution of the disease in each country, thereby improving public policies for mitigating or suppressing the effects of COVID-19 on society ahead of obtaining a vaccine. Our new methodology proves more effective in explaining the evolution of COVID-19 worldwide than traditional growth functions, including highlighting several inflection points and regime-switching moments. Moreover, results from this research can be used by managers, for example, to provide an econometric justification for the prioritizing of vaccination programmes in the health care sector.

The use of GAM functions to predict the confirmed cases and deaths prove adequate, even with the occurrence of spikes or “second waves” in these series. Our new approach even allows the identification of several inflection points throughout the daily confirmed cases and deaths series: an advance when compared to traditional growth functions.

However, we recognize that for monitoring pandemics and epidemic outbreaks in the early stages, growth functions are still important for this purpose, as demonstrated by [18]. This brings us to the main limitation of our evaluation framework: the size of the epidemiological time series. It was empirically verified that the smallest size for the proper use of GAM functions and Markov-Switching models is 60 observations.

We incorporate new evidence that pandemic fatigue is taking hold, especially after the start of vaccination. This decrease in commitment to fighting the pandemic alters the behavior of the forecast errors present in the COVID-19 curves, causing a structural break in the variance of the residuals, or forecast errors. This allows us the opportunity to take advantage of Markov-Switching (MS) models, built on the residuals of our forecasts, to specifically identify this behavior using regime-switching in the COVID-19 time series. The application of the Markov-Switching models in the residuals of the GAM functions proves to be viable concerning the identification of structural breaks in these series, effectively pointing to a prevalence of the MSwM models for the confirmed cases, and the MSGARCH for deaths. Besides, when the MSGARCH is chosen, the prevalent model indicates that the variance in the eGARCH model responds asymmetrically to rises and falls in COVID-19 numbers.

Finally, our new framework for assessing the effectiveness of countries in controlling the spread of COVID-19 in their territories, as well as the number of deaths, provides a new lens for visualizing and understanding the world panorama, helping to identify the countries with the most effective strategies, and even allowing additional new explanatory variables to be used in the y-axis, such as the death rate from infected people. The new outcomes presented in this research will allow key stakeholders to check whether or not public policies and interventions in the fight against COVID-19 are having an effect. We can easily identify examples of best practice and promote such policies more widely around the world. Not least, the application of our evaluation framework to the vaccine dataset developed by [56] is our main recommendation for future studies.

## Supplementary Information

**Additional file 1** Script1.

**Additional file 2** Script2.

**Additional file 3** Script3.

**Additional file 4** List of countries’ abbreviations.

## Data Availability

Countries’ data were collected using tidycovid19 R package [6]. Once the package is installed, public access to the database is free and open. To access the link for the global database of COVID-19 vaccinations, see [56]. Besides, all codes written in R used in this study (Additional file 1, Additional file 2, and Additional file 3) are available in supplementary material, as well as a list of countries abbreviations (Additional file 4), to promote and disseminate our findings widely.

## Abbreviations

CSSE: Centre for Systems Science and Engineering
GAM: Generalized Additive Models
REML: restricted maximum likelihood
ML: maximum likelihood
GCV: generalized cross-validation
RRSE: Root Relative Squared Error
WHO: World Health Organization
MSwM: pure Markov-Switching
MSGARCH: Markov-Switching Generalized Autoregressive Conditional Heteroskedasticity
GED: generalized error distribution
PAM: Partitioning Around Medoids
ASW: Average Silhouette Width
