# Supplementary material for: Using GAM functions and Markov-Switching models in an evaluation framework to assess countries’ performance in controlling the COVID-19 pandemic
Source: BMC Public Health. 2021 Nov 27;21:2173. doi: 10.1186/s12889-021-11891-6 (PMC8626735; doi:10.1186/s12889-021-11891-6)
Supplement: Supplementary file 4 — Additional file 4 List of countries’ abbreviations. [file 12889_2021_11891_MOESM4_ESM.pdf]

List of countries' abbreviations

Country	iso3c	Country	iso3c
Afghanistan	AFG	Czechia	CZE
Albania	ALB	Democratic Republic of Congo	COD
Algeria	DZA	Denmark	DNK
Andorra	AND	Djibouti	DJI
Angola	AGO	Dominica	DMA
Anguilla	AIA	Dominican Republic	DOM
Antigua and Barbuda	ATG	Ecuador	ECU
Argentina	ARG	Egypt	EGY
Armenia	ARM	El Salvador	SLV
Aruba	ABW	Equatorial Guinea	GNQ
Australia	AUS	Estonia	EST
Austria	AUT	Eswatini	SWZ
Azerbaijan	AZE	Ethiopia	ETH
Bahamas	BHS	Faeroe Islands	FRO
Bahrain	BHR	Fiji	FJI
Bangladesh	BGD	Finland	FIN
Barbados	BRB	France	FRA
Belarus	BLR	French Polynesia	PYF
Belgium	BEL	Gabon	GAB
Belize	BLZ	Gambia	GMB
Benin	BEN	Georgia	GEO
Bermuda	BMU	Germany	DEU
Bhutan	BTN	Ghana	GHA
Bolivia	BOL	Gibraltar	GIB
Bonaire Sint Eustatius and Saba	BES	Greece	GRC
Bosnia and Herzegovina	BIH	Greenland	GRL
Botswana	BWA	Grenada	GRD
Brazil	BRA	Guatemala	GTM
British Virgin Islands	VGB	Guernsey	GGY
Brunei	BRN	Guinea	GIN
Bulgaria	BGR	Guinea-Bissau	GNB
Burkina Faso	BFA	Guyana	GUY
Cambodia	KHM	Honduras	HND
Cameroon	CMR	Hong Kong	HKG
Canada	CAN	Hungary	HUN
Cape Verde	CPV	Iceland	ISL
Cayman Islands	CYM	India	IND
Central African Republic	CAF	Indonesia	IDN
Chile	CHL	Iran	IRN
China	CHN	Iraq	IRQ
Colombia	COL	Ireland	IRL
Comoros	COM	Isle of Man	IMN
Congo	COG	Israel	ISR
Costa Rica	CRI	Italy	ITA
Cote d'Ivoire	CIV	Jamaica	JAM
Croatia	HRV	Japan	JPN
Cuba	CUB	Jersey	JEY
Curacao	CUW	Jordan	JOR
Cyprus	CYP	Kazakhstan	KAZ

List of countries' abbreviations

Country	iso3c	Country	iso3c
Kenya	KEN	Peru	PER
Kiribati	KIR	Philippines	PHL
Kuwait	KWT	Poland	POL
Kyrgyzstan	KGZ	Portugal	PRT
Laos	LAO	Qatar	QAT
Latvia	LVA	Romania	ROU
Lebanon	LBN	Russia	RUS
Lesotho	LSO	Rwanda	RWA
Liberia	LBR	Saint Kitts and Nevis	KNA
Libya	LBY	Saint Lucia	LCA
Liechtenstein	LIE	Saint Vincent and the Grenadines	VCT
Lithuania	LTU	Samoa	WSM
Luxembourg	LUX	San Marino	SMR
Macao	MAC	Sao Tome and Principe	STP
Madagascar	MDG	Saudi Arabia	SAU
Malawi	MWI	Senegal	SEN
Malaysia	MYS	Serbia	SRB
Maldives	MDV	Seychelles	SYC
Mali	MLI	Sierra Leone	SLE
Malta	MLT	Singapore	SGP
Mauritania	MRT	Sint Maarten (Dutch part)	SXM
Mauritius	MUS	Slovakia	SVK
Mexico	MEX	Slovenia	SVN
Moldova	MDA	Solomon Islands	SLB
Monaco	MCO	Somalia	SOM
Mongolia	MNG	South Africa	ZAF
Montenegro	MNE	South Korea	KOR
Montserrat	MSR	South Sudan	SSD
Morocco	MAR	Spain	ESP
Mozambique	MOZ	Sri Lanka	LKA
Myanmar	MMR	Sudan	SDN
Namibia	NAM	Suriname	SUR
Nauru	NRU	Sweden	SWE
Nepal	NPL	Switzerland	CHE
Netherlands	NLD	Syria	SYR
New Caledonia	NCL	Taiwan	TWN
New Zealand	NZL	Tajikistan	TJK
Nicaragua	NIC	Thailand	THA
Niger	NER	Togo	TGO
Nigeria	NGA	Tonga	TON
North Macedonia	MKD	Trinidad and Tobago	TTO
Northern Cyprus	OWID_CYN	Tunisia	TUN
Norway	NOR	Turkey	TUR
Oman	OMN	Turks and Caicos Islands	TCA
Pakistan	PAK	Tuvalu	TUV
Palestine	PSE	Uganda	UGA
Panama	PAN	Ukraine	UKR
Papua New Guinea	PNG	United Arab Emirates	ARE
Paraguay	PRY	United Kingdom	GBR

List of countries' abbreviations

Country	iso3c	Country	iso3c
United States	USA		
Uruguay	URY		
Uzbekistan	UZB		
Vanuatu	VUT		
Venezuela	VEN		
Vietnam	VNM		
Wallis and Futuna	WLF		
Yemen	YEM		
Zambia	ZMB		
Zimbabwe	ZWE		
